# A novel immune-related risk-scoring system associated with the prognosis and response of cervical cancer patients treated with radiation therapy

**DOI:** 10.3389/fmolb.2023.1297774

**Published:** 2023-11-10

**Authors:** Zhuna Wu, Qiuya Lin, Liying Sheng, Weihong Chen, Meili Liang, Danni Wu, Yumin Ke

**Affiliations:** ^1^ Department of Gynecology and Obstetrics, The Second Affiliated Hospital of Fujian Medical University, Quanzhou, China; ^2^ Department of Operation, The Second Hospital of Jinjiang, Quanzhou, China

**Keywords:** tumor microenvironment, raidotherapy, cervical cancer, risk score model, tumor-infiltrating immune cells

## Abstract

**Objective:** The tumor microenvironment plays a critical role in the radiotherapy and immunotherapy response of cervical squamous cell carcinoma and endocervical adenocarcinoma (CESC). Radioresistance is a key factor in treatment failure among patients who receive radical radiotherapy. Thus, new immune-related biomarkers associated with radiotherapy response in CESC are needed.

**Methods:** In this study, the CIBERSORT and ESTIMATE methods were applied to determine the percentage of tumor-infiltrating cells and the number of immune components in 103 CESCs treated with radiotherapy from The Cancer Genome Atlas (TCGA) database. The main dysregulated genes were subjected to multivariate and univariate analyses. The prognostic value of this system was studied via receiver operating characteristic curve and survival analysis. For further confirmation, the biomarkers’ expression levels and predictive value were validated by immunohistochemistry (IHC) and qRT-PCR. The CIBERSORT algorithm was used to calculate the compositional patterns of 22 types of immune cells in cervical cancer patients treated with radiation therapy.

**Results:** Data for 17 radioresistant and 86 radiosensitive tumors were obtained from the The Cancer Genome Atlas database. 53 immune-related DEGs were identified. GO and KEGG analyses revealed that the DEGs were enriched in protein kinase B signaling, growth factors in cytokines, the MAPK pathway and the PI3K-Akt pathway. Then, 14 key immune-related genes built a risk scoring model were deemed prognostic in CESC with radiotherapy. The area under the curve (AUC) of the model was 0.723, and the high-risk group presented worse outcomes than the low-risk group. In addition, the high-risk group tended to have persistent tumors (*p* = 0.001). The high expression of WT1 and SPOUYT4 were associated with relapse, the high expression of Angiotensinogen and MIEN1 were associated with nonrelapse. Analysis of the immune microenvironment indicated that M0 macrophages, M2 macrophages, activated mast cells and resting memory CD4^+^ T cells were positively correlated with the risk score (*p* < 0.05).

**Conclusion:** The novel immune-related risk scoring system has some advantages in predicting the prognosis and treatment response of cervical cancer patients treated with radiotherapy. Moreover, it might provide novel clues for providing targeted immune therapy to these patients.

## 1 Introduction

Cervical cancer (CC) is the first incidence of female neoplasms in 23 countries worldwide, and also the first death of female neoplasms in 36 countries worldwide (Sung et al., 2021), with the number of incidence and deaths located in the fourth place of female neoplasms worldwide, accounting for 6.5% of the total number of incidence of female neoplasms and 7.7% of the total number of deaths, respectively (Sung et al., 2021). At present, CC is still the most common malignant tumour among female genital tract tumours, with 40–59 years of age as the high incidence age group (Li et al., 2017b), and showing a trend towards a younger age group. The mainstay of treatment for CC is surgery and radiotherapy, with about 80% of patients requiring radiotherapy, especially those in advanced stages. With the popularity of CC screening methods and vaccines, although the prognosis of early-stage CC patients is good, with a 5-year survival rate as high as 92%, more than half of the patients are diagnosed with progressive stage, and all patients with progressive stage need radiotherapy, and the 5-year survival rate decreases rapidly to only 16.8% (Liontos et al., 2019), of which insensitivity to radiotherapy is one of the main reasons for treatment failure. Although current research on radiation resistance is emerging, there is no effective method to predict or effectively reverse resistance in CC. Therefore, studies regarding how to predict and solve the problem of radiotherapy resistance are needed. In addition, the immune-related cells and molecular mechanisms related to radiation-resistant CC are unclear and have not been included in the currently recognized risk scoring system, which makes it difficult to study the relevant immune mechanisms of radiation-resistant cervical cancer.

The tumor microenvironment (TME), which includes factors such as tumor-infiltrating lymphocytes, is closely related to the biological behavior of tumors and tumor prognosis (Fridman et al., 2012). Immunotherapies such as checkpoint inhibitors and monoclonal antibodies can regulate the immune function of the body by changing the tumor microenvironment and generating an antitumor immune response. The role of the abnormal expression of tumor immune-related genes in tumor immune escape is becoming a new direction in tumor research. The abnormal immunogenomic expression has a major influence on the prognosis of non-small-cell lung cancer, ovarian cancer and gastric cancer (Li et al., 2017a; Shen et al., 2019; Yang et al., 2019). For a long time, it has been believed that local radiotherapy exerts a direct killing effect on cancer cells by inducing DNA damage. Recently, some researchers have shown that the “field” effect of radiotherapy is also dependent on the immune system and involves promoting tumor antigen amplification, altering gene expression, activating the immune response, inducing the T cell response, and regulating tumor stromal cells. Chen et al. (Chen et al., 2021) focuses on the mechanism of radiotherapy and T cells, as well as related to research progress in theusage of PD-1/PD-L1. Patients who received radio-immunotherapy showed that reduction of Ter-cells, artemin, and GFRα3, an artemin signaling partner, were related to tumor regression (Hou et al., 2021). One study showed that radiotherapy combined with anti-PD-1 or anti-Treg treatment also increased the tumor control rate (Sharabi et al., 2015). Twyman et al. (Twyman-Saint Victor et al., 2015) used a single 20-Gy dose of radiotherapy combined with an anti-CTLA-4 antibody in a rat model of melanoma. The combination therapy was observed to have a significant effect, but therapeutic tolerance was also noted. Further study showed that tumor cell surface PD-L1 upregulation leading to T cell dysfunction was the main tolerance mechanism. Therefore, anti-PD-L1 therapies should be combined with radiotherapy and anti-CTLA-4 antibody treatment in tolerant patients, as antibodies can regulate the function of effector T cells and enhance the curative effect. However, the effect of abnormal immune genome expression on the prognosis of patients with cervical cancer radiotherapy resistance has not been reported.

Herein, to further explore the immune-related characteristics and mechanism of cervical cancer radiotherapy resistance, we explored the differentially expressed genes (DEGs) between cervical squamous cell carcinoma and endocervical adenocarcinoma (CESC) samples stratified by immune infiltration level and radiotherapy response and established a risk prediction system for cervical cancer radiotherapy resistance that reflects the alteration of TME in CESC treated with radiotherapy. With this strategy, we aimed to provide a new option for the selection of individualized immune-related treatments for radioresistant CESC.

## 2 Materials and methods

### 2.1 Data collection and enrichment analysis

Transcriptome RNA-seq data of 105 CESC cases (86 radiosensitive cases; 17 radioresistant cases) and the matching clinical-pathological data were downloaded from the TCGA CESC dataset. For each sample, the percentage of immune components in the TME was evaluated, and the ESTIMATE algorithm was applied with the R language (version 3.6.3) and the ESTIMATE package (Team, 2018)to determine the ImmuneScore, which is positively correlated with the percentage of immune cells, implying that a high respective score is directly proportional to a high percentage of the matching component in the TME. According to the median ImmuneScore, the 105 cases were separated into high- or low-score groups. The differential expression of genes between the high score group with the low-score group was analyzed using the Limma package. DEGs with |log foldchange (FC)| > 1 and false discovery rate (FDR) < 0.05 were considered significant. Fifty-three DEGs were analyzed with R language with the clusterProfiler, enrichplot, and ggplot2 packages via GO and KEGG analyses. The results with *p* < 0.05 were considered significantly enriched.

### 2.2 Survival analysis and risk score system

A total of 307 patients were retained for the survival analysis, with follow-up times ranging from 0 to 16.3 years. We employed univariate analysis to screen genes that were related to survival (*p* < 0.05). Independent prognostic genes were selected by establishing a multivariate Cox hazards regression model. A regression coefficient (β) was obtained from the multivariate Cox proportional hazards regression for each gene; then, we added the products of the gene expression values multiplied by the β values to construct a prognostic gene signature. The following formula was used: risk score = expression of gene1 × β1gene1 + … expression of genen × βngenen (Lin et al., 2018). According to the median risk score, patients were separated into two groups (low-risk or high-risk). To assess the ability of the model to predict survival, we performed ROC curve analysis with the R packages and calculated the area under the curve (AUC).

### 2.3 TIC profile

For all tumor samples, we used the CIBERSORT computational method to estimate the TIC abundance profile, and then only cancer samples (*p* < 0.05) were selected by quality filtering for subsequent analysis.

### 2.4 Patient and tissue samples

Sixty-nine paraffin-embedded cervical cancer patients treated with radiotherapy specimens were diagnosed at The Second Affiliated Hospital of Fujian Medical University (Fujian, China) from June 2019 to December 2022. The main treatment of all patients underwent radiotherapy. The research was approved by the Research Ethics Committee of The Second Affiliated Hospital of Fujian Medical University prior to the study.

### 2.5 Immunohistochemistry (IHC)

IHC staining was operated as previously described (Chen et al., 2016). The primary antibodies included anti-WT1 (Servicebio, Wuhan), anti-AGT (Servicebio, Wuhan), anti-MIEN1 (Bioss, Beijing) and anti-SPOUYT4 (Bioss, Beijing). The proportion of immunostaining intensity was scored as follows: 0 = negative; 1 = light yellow; 2 = brownish yellow; 3 = or tan. The immunostaining was scored as follows: 1 = less than 1/3; 2 = between 1/3 and 2/; 3 = more than 2/3. The final score for biomarkers expression were calculated by multiplying the 2 scores. The slides were classified to low and high expression group, relative to scores of <3 or ≥3, respectively. The histopathological diagnosis of the patients was established by two pathologists specialized in Gynecologic Oncology in our study.

### 2.6 Quantitative real-time PCR

Total RNA was extracted from frozen CC tissue utilizing Tirzol (TaKaRa, Japan), and then, cDNA was prepared according to the protocol (TaKaRa, Japan). The detailed procedure is presented in the Supplementary Methods. GAPDH was used as an internal reference, relative mRNA expression of WT1, AGT, MIEN1 and SPOUTY4 was calculated by the 2^−ΔΔCT^ method. qRT-PCR for each sample was repeated in three independent experiments. The primer sequences are shown below:

GAPDH

Forward: 5′- GAA​CGG​GAA​GCT​CAC​TGG-3′,

Reverse: 5′- GCC​TGC​TTC​ACC​ACC​TTC​T -3′.

WT1

Forward: 5′- GGG​TAC​GAG​AGC​GAT​AAC​CAC -3′

Reverse: 5′- CCG​TGC​GTG​TGT​ATT​CTG​TAT​TG -3’.

AGT

Forward: 5′- CCC​ACG​CTC​TCT​GGA​CTT​CA-3′,

Reverse: 5′- GGA​CGT​AGG​TGT​TGA​AAG​CCA -3′.

MIEN1

Forward: 5′- CAG​GGT​AAG​TGC​CCA​CGA​A-3′

Reverse: 5′- TAC​TGC​CAA​TAG​CTG​ACA​TTG​C -3′.

SPOUTY4

Forward: 5′- AGT​GAG​TCC​AAG​CCT​CGT​TCC-3′,

Reverse: 5′- CCC​GAG​AGT​TAG​TTG​ATG​CAG​TT -3′.

### 2.7 Statistical analysis

We used R software (v.3.6.3) for all statistical analyses. We applied the Wilcoxon signed-rank test, Kolmogorov-Smirnov test and logistic regression to analyze the relation of the risk score to clinicopathological characteristics. For survival analysis, we utilized the Cox multivariate system and the Kaplan-Meier method. The median value was used as the cutoff value of the risk score. Unpaired *t*-test was used as described above. Differences with *p* < 0.05 were considered statistically significant for all statistical analyses, unless otherwise specified.

## 3 Results

### 3.1 Study procedure

**FIGURE 1:**
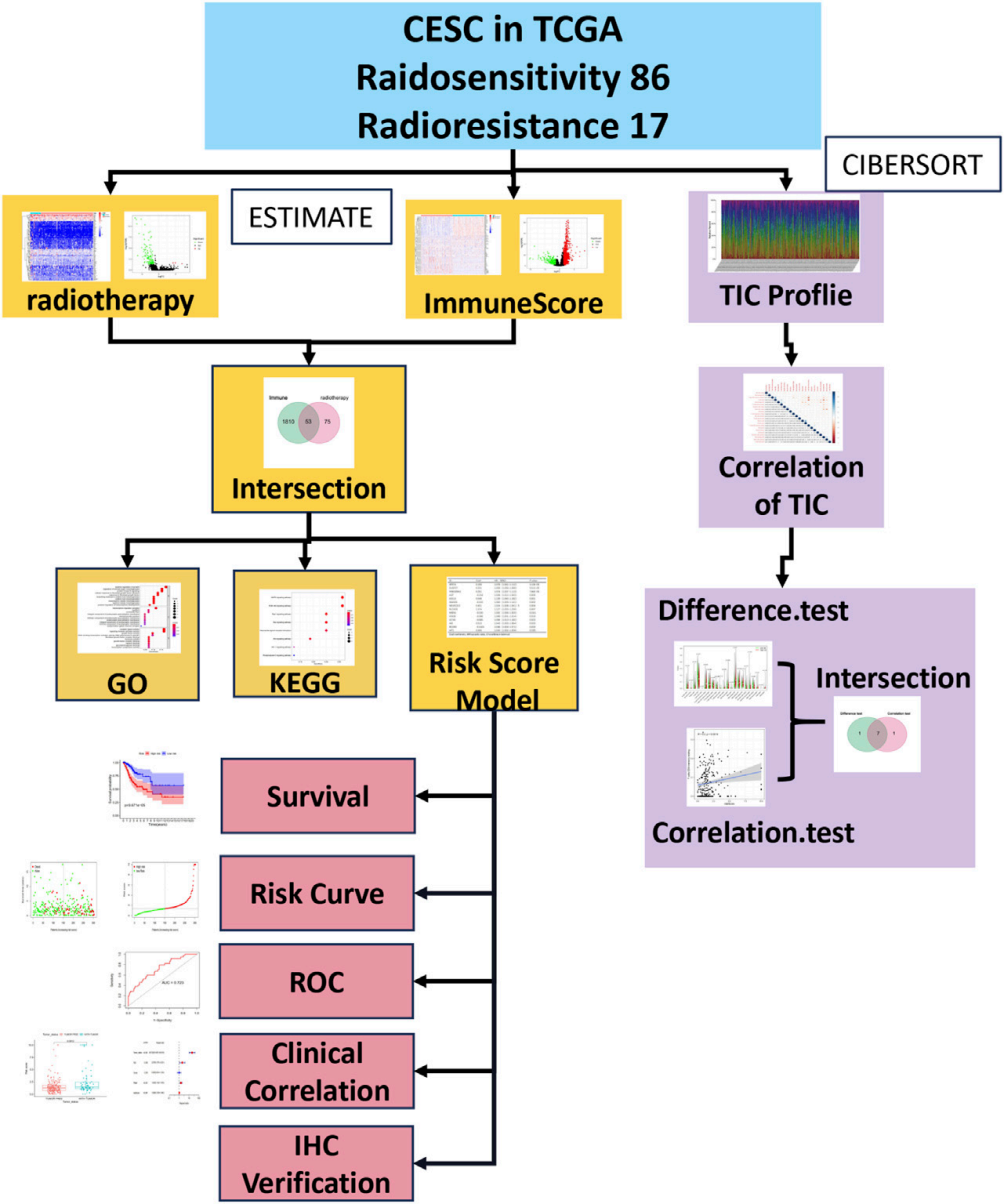
Analysis workflow of this study.

We show the analysis procedure of our study in Figure 1. In CESCs treated with radiotherapy, we computed the percentage of TICs and the number of immune components. We downloaded the transcriptome RNA-seq data of 105 cases from the TCGA database and then quantified them with CIBERSORT and ESTIMATE algorithms. We conducted GO and KEGG analyses on DEGs that were common in the ImmuneScore and radiotherapy response comparisons. According to the Cox proportional hazards model, we conducted univariate and multivariate survival analyses to identify key prognostic genes. Key prognostic genes were filtered to construct the risk scoring system, and the optimal system was found according to the Akaike information criterion (AIC). We concentrated on the risk score system for the following analysis, which included survival and clinicopathological characteristic correlation analysis, Cox regression analysis, risk curves, and analysis of correlations with TICs. The biomarkers’ expression levels and predictive value in cervical cancer were validated by IHC.

### 3.2 Common DEGs from the ImmuneScore and radiotherapy response comparison analyses in CESC patients were mainly immune-related genes

**FIGURE 2:**
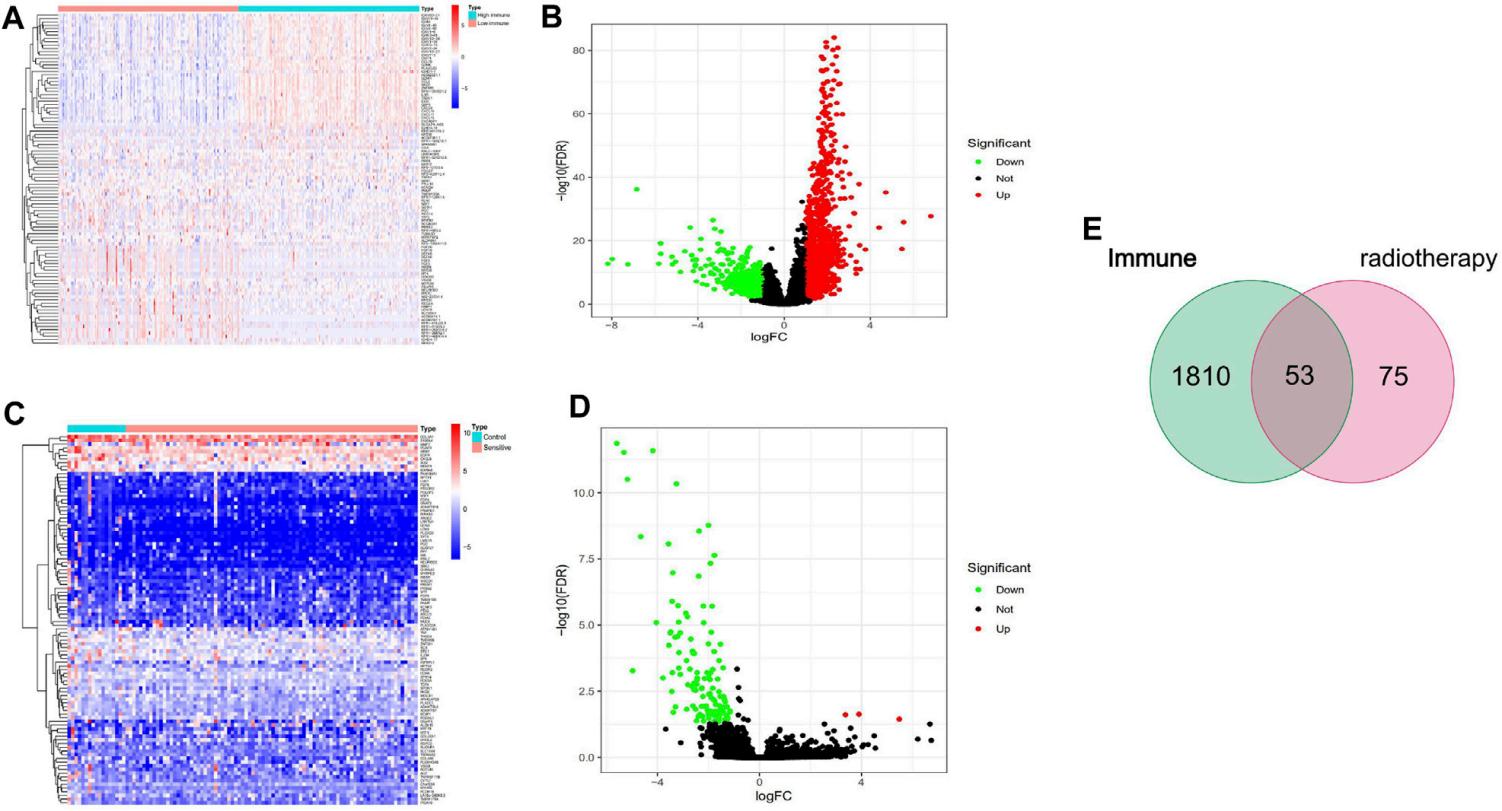
Identifcation of DEGs. **(A, B)** volcano and Heatmap plots of 1863 DEGs generated by comparison of the high score group vs. the low score group in ImmuneScore in cervical cancer with radiotherapy from TCGA database. **(C, D)** volcano and Heatmap plots of 128 DEGs in radioresistance and radiosensitivity cervical cancer from TCGA database. The red dots in the volcano plots represent upregulation, the green dots represent downregulation and black dots represent genes without differential expression. Row name of heatmap is the gene name, and column name is the ID of samples which not shown in plot.The colors from red toblue represent expression level from high to low in the heatmaps. **(E)** Venn plot showing common 53 DEGs shared by ImmuneScore and Radiotherapy.

**FIGURE 3:**
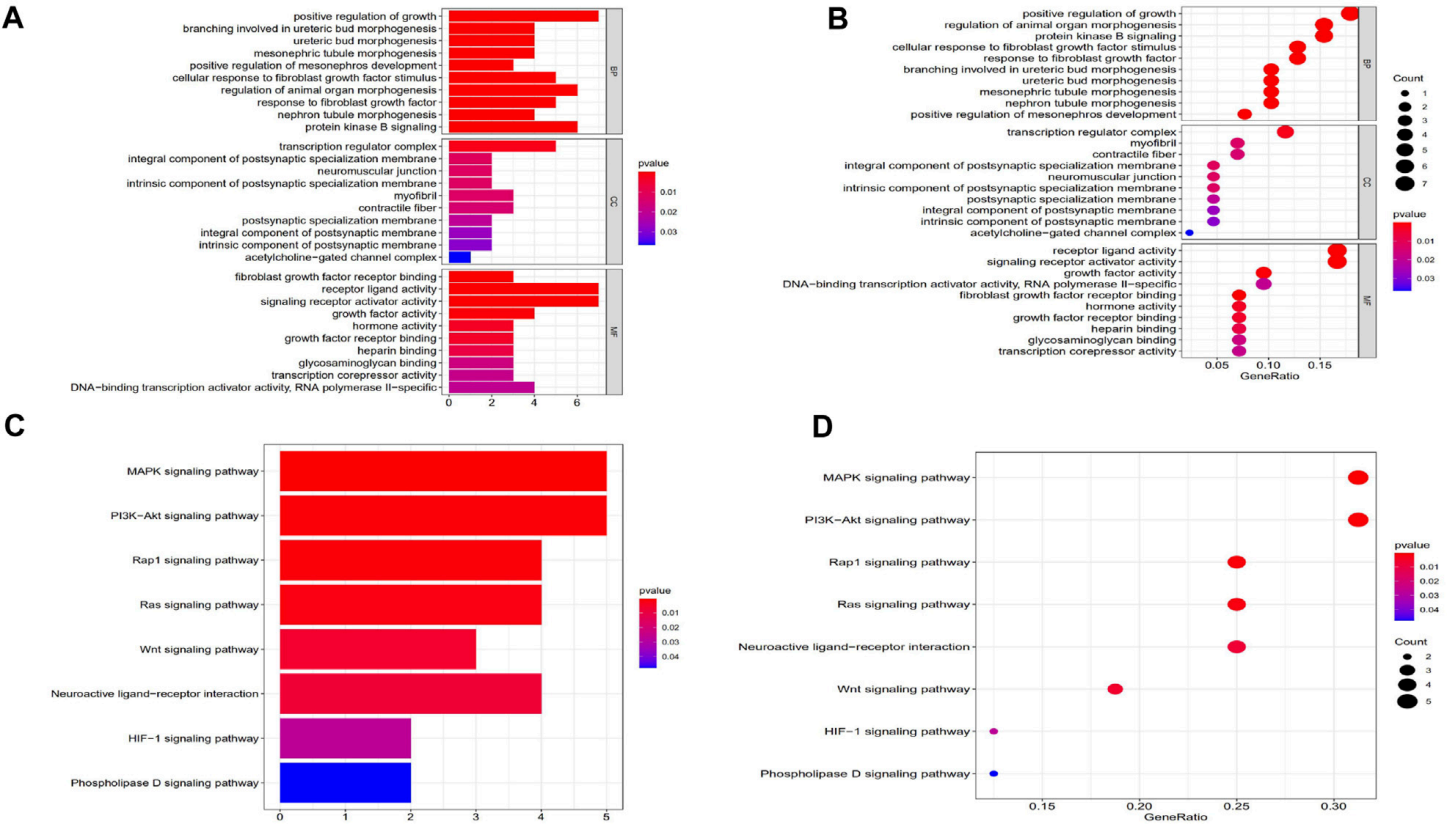
GO and KEGG enrichment analysis of DEGs. **(A, B)** GO analysis. GO analysis divided DEGs into three functional groups: molecular function (MF), biological processes (BP), and cervical cancer (CC). **(C, D)** KEGG analysis of DEGs.

To assess changes in the TME based on the immune response and radiotherapy, we assessed the modification of the gene profiles of CESC patients treated with radiotherapy. We obtained a total of 1863 DEGs when comparing patients with high *versus* low ImmuneScores (according to the median) (Figures 2A,B). In total, 128 DEGs were obtained when comparing patients who responded to radiotherapy and those who did not (Figure 2C,D). A total of 53 genes were common to both the ImmuneScore and radiotherapy analysis, as shown in the Venn diagram (Figure 2E). To determine the potential function of DEGs, functional enrichment analysis was performed for the intersecting genes related to immune score and radiotherapy. The results from the GO analysis showed that the DEGs were closely related to tumor-related GO terms, such as positive regulation of growth and protein kinase B and to immune-related GO terms, particularly growth factors in cytokines, receptor ligand activity, and receptor activator activity (*p* < 0. 05, Figures 3A,B). In addition, KEGG analysis showed enrichment of the MAPK signaling pathway and PI3K-Akt signaling pathway (*p* < 0. 05, Figures 3C,D), which are significantly related to tumor growth, drug resistance, and immune factors (cytokines). Therefore, the overall function of DEGs appeared to be related to tumor therapy- and immune-related activities, suggesting that immune components may be a primary feature of the TME that affects CESC radiotherapy response.

### 3.3 Establishment of a risk scoring system

**FIGURE 4:**
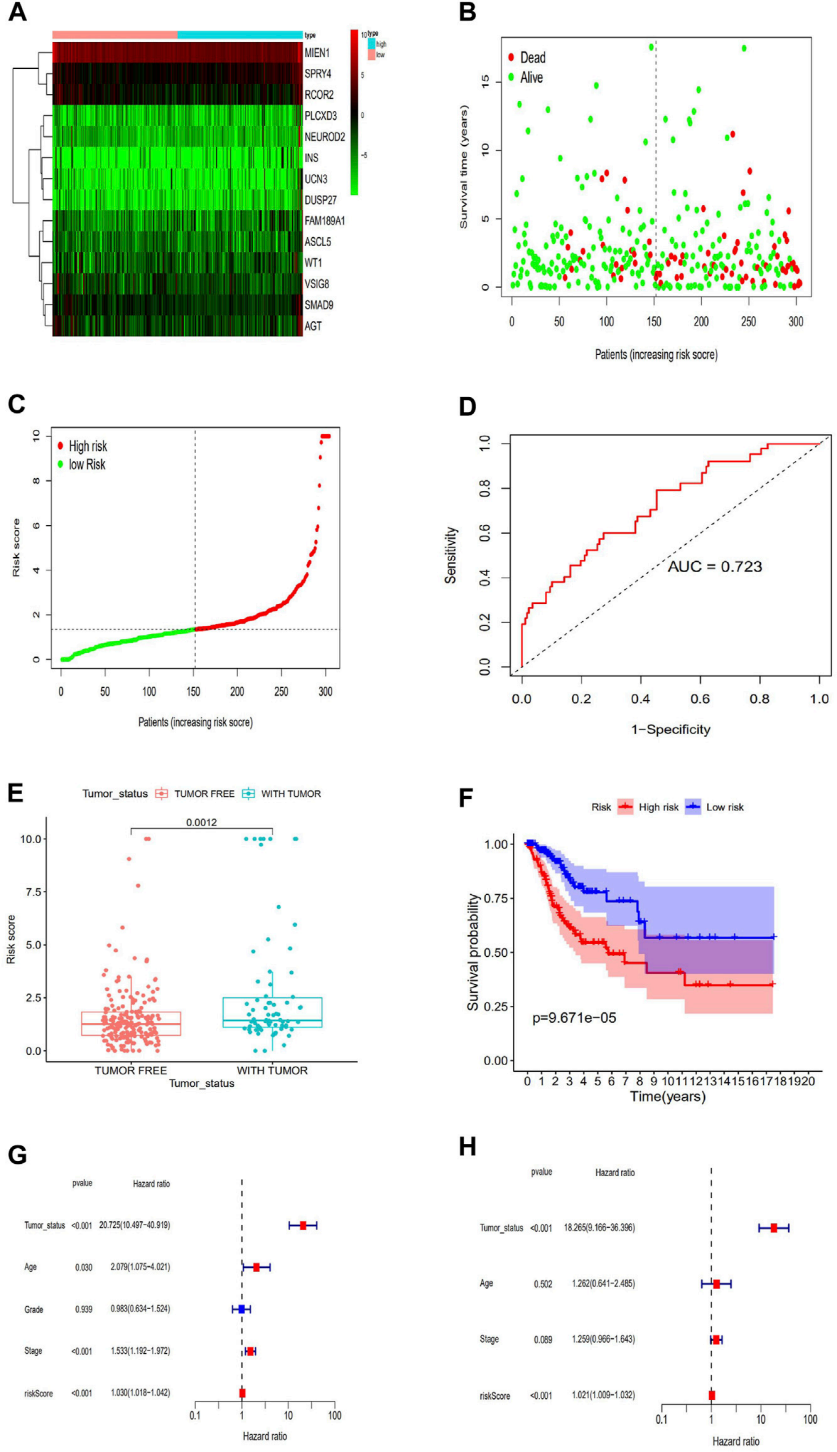
Establishment of a risk scoring system. **(A)**Risk score estimated of each patient on the basis of 14 hub genes expression. **(B)**ROC curve for the risk model. **(C)**The distributions of OS status, OS and risk score.Patients were divided into high risk group and low risk group. Survival analysis of the patients is also shown. **(D)**The distribution and median value of the risk scores in the TCGA cohort. **(E)**Kaplan-Meier curves for the OS of patients in the high-risk group and low-risk group. **(F)**Association between the risk model and Tumor_status after radiotherapy. **(G)**Validation of the prognostic value of the risk model---Univariate regression model. **(H)**Validation of the prognostic value of the risk model---Multi-cox hazards regression model.

Concerning the Cox proportional hazards system, we conducted univariate and multivariate survival analyses to choose key prognostic genes. According to the AIC, to find the optimal system, we selected the genes with *p* < 0.05. A risk score algorithm consisting of 14 key genes (SPRY4, MIEN1, PLCXD3, FAM189A1, WT1, SMAD9, INS, UCN3, RCOR2ASCL5, AGT, DUSP27, VSIG8, NEUROD2) was established (Table 1). Six of these genes (MIEN1, SMAD9, UCN3, RCOR2, AGT VSIG8) were negatively related to survival, while the other eight genes were positively associated with survival. According to the expression of the 14 key genes, we estimated the risk score for each patient (Figure 4A). A ROC curve was built to evaluate the predictive accuracy, and the AUC was 0.723 (Figure 4B). All patients scored were classified into two groups via the risk scoring system: the high-risk group or the low-risk group (Figures 4C,D). Likewise, the high-risk group had worse outcomes than the low-risk group according to the survival analysis (*p* < 0.001) (Figure 4E). In addition, we also explored the clinical significance of the risk scoring system. The results showed that the risk scoring system was related to tumor status after radiotherapy (*p* < 0.05) but not to age, differentiation degree or tumor-node-metastasis (TNM) stage (*p* > 0.05) (Figure 4F). To further verify whether the risk scoring system could independently predict the prognosis of patients CESC receiving radiotherapy, we performed survival analysis via univariate and multivariate Cox hazards regression. We discovered that tumor status (with tumor vs tumor-free, HR = 18.26, *p* < 0.001) and risk score (high vs low, HR = 1.021, *p* < 0.001) were independent prognostic factors in the multivariate analysis (Figures 4G,H).

**TABLE 1:** Prognostic risk model for cervical cancer with radiotherapy.

	Coef	HR; 95%CI	*p*-value
SPRY4	0.193	1.076 (1.041–1.112)	1.13E-05
DUSP27	0.331	1.059 (1.030–1.090)	5.51E-05
FAM189A1	0.261	1.074 (1.037–1.113)	7.66E-05
AGT	−0.216	1.028 (1.012–1.043)	0.000
ASCL5	0.546	1.109 (1.040–1.182)	0.001
SMAD9	−0.533	1.083 (1.029–1.141)	0.002
NEUROD2	0.401	1.024 (1.008–1.041)	0.004
PLCXD3	1.574	1.127 (1.033–1.230)	0.007
MIEN1	−0.030	1.002 (1.000–1.003)	0.015
VSIG8	−0.066	1.008 (1.001–1.014)	0.018
UCN3	−0.685	1.094 (1.013–1.182)	0.023
INS	0.513	1.043 (1.003–1.084)	0.033
RCOR2	−0.1421	1.036 (1.003–1.071)	0.033
WT1	0.060	1.030 (1.002–1.059)	0.035

*Coef* coefcients, *HR*, hazards ratio; *CI*, confdence interval.

### 3.4 Validation of a risk scoring system

**FIGURE 5:**
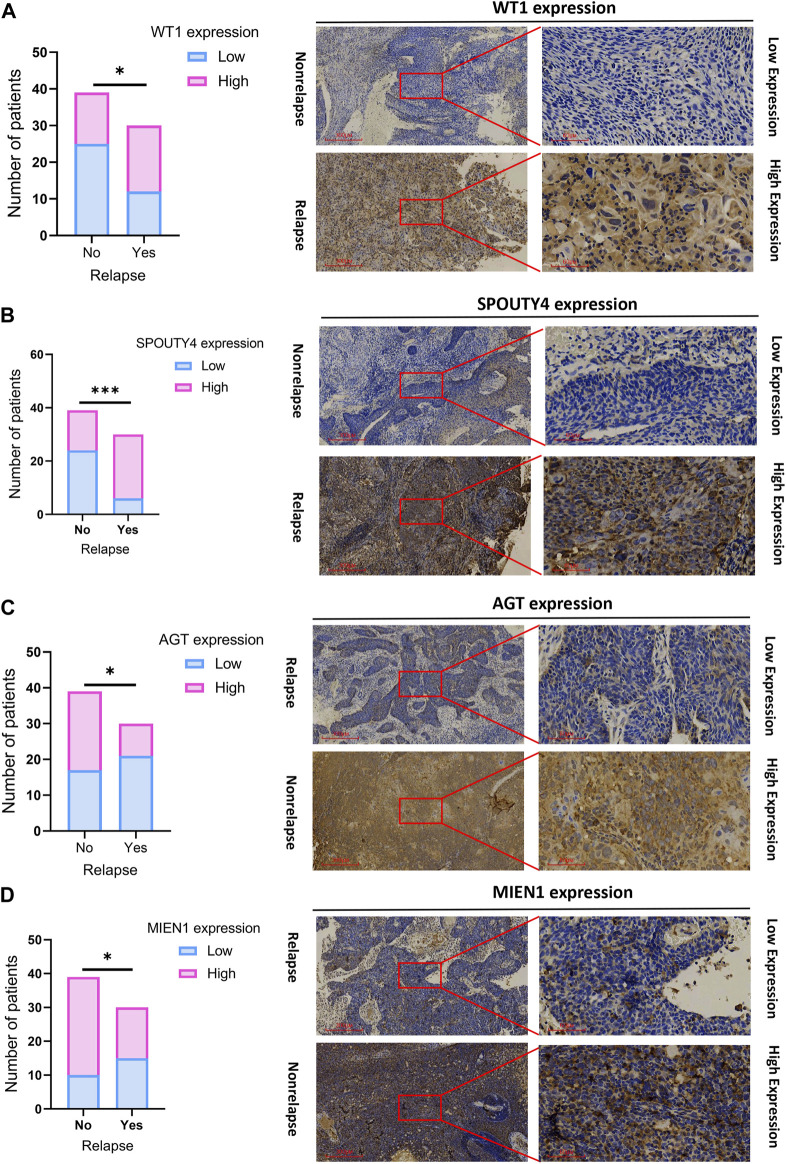
Validation of a risk scoring system (WT1, AGT, MIEN1 and SPOUTY4). **(A)** Significantly high WT1 expression was observed in cervical caner tissues with relapse. Representative images (×40 and ×200) of IHC staining for WTI in 69 patients (high expression vs low expression). **(B)** Significantly high SPOUTY4 expression was observed in cervical cancer tissues with relapse specimens. Representative images (×40 and ×200) of IHC staining for SPOUTY4 in 69 cervical cancer patients (high expression vs low expression). **(C)** Significantly high AGT expression was observed in cervical cancer tissues with nonrelapse specimens. Representative images (×40 and ×200) of IHC staining for AGT in 69 cervical cancer patients (high expression vs low expression). **(D)** Significantly high MIENI expression was observed in cervical cancer tissues with nonrelapse specimens. Representative images (×40 and ×200) of IHC staining for MIEN1 in 69 cervical cancer patients (high expression vs low expression). Scale bars are shown. *p < 0.05. *p* values were calculated by chi-square tests.

**FIGURE 6:**
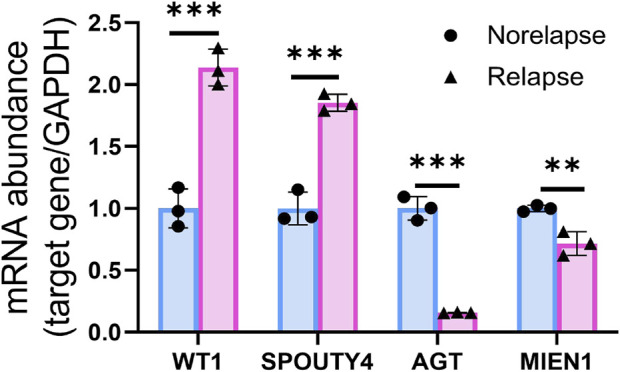
qRT-PCR of WT1, AGT, MIEN1 and SPOUTY4 expression in nonrelapse CC specimens when compared with relapse CC specimens.

We assessed the expression of WT1, AGT, MIEN1 and SPOUTY4 across cervical cancer tissues via IHC. To determine the clinical importance of WT1, AGT, MIEN1 and SPOUTY4 in CC, we detected WT1, AGT, MIEN1 and SPOUTY4 protein levels in 69 samples from CC patients who underwent radiotherapy as a primary treatment regimen (Supplementary Table S1,S2,S3,S4). We found that high expression of WT1 and SPOUTY4 were associated with relapse (Figures 5A, 6B; *p* < 0.05), high expression of AGT and MIEN1 were associated with nonrelapse (Figures 5C,D; *p* < 0.05). For further clinical validation, we assessed the expression of WT1, AGT, MIEN1 and SPOUTY4 using qRT-PCR and revealed that low expression of AGT and MIEN1 were associated with relapse, conversely, high expression of WT1 and SPOUTY4 were associated with relapse (Figure 6; *p* < 0.05). The above results indicating that the WT1, AGT, MIEN1 and SPOUTY4 had a higher predicitive capacity to radiosensitivity.

### 3.5 Correlation of the risk scoring system with the percentage of TICs

**FIGURE 7:**
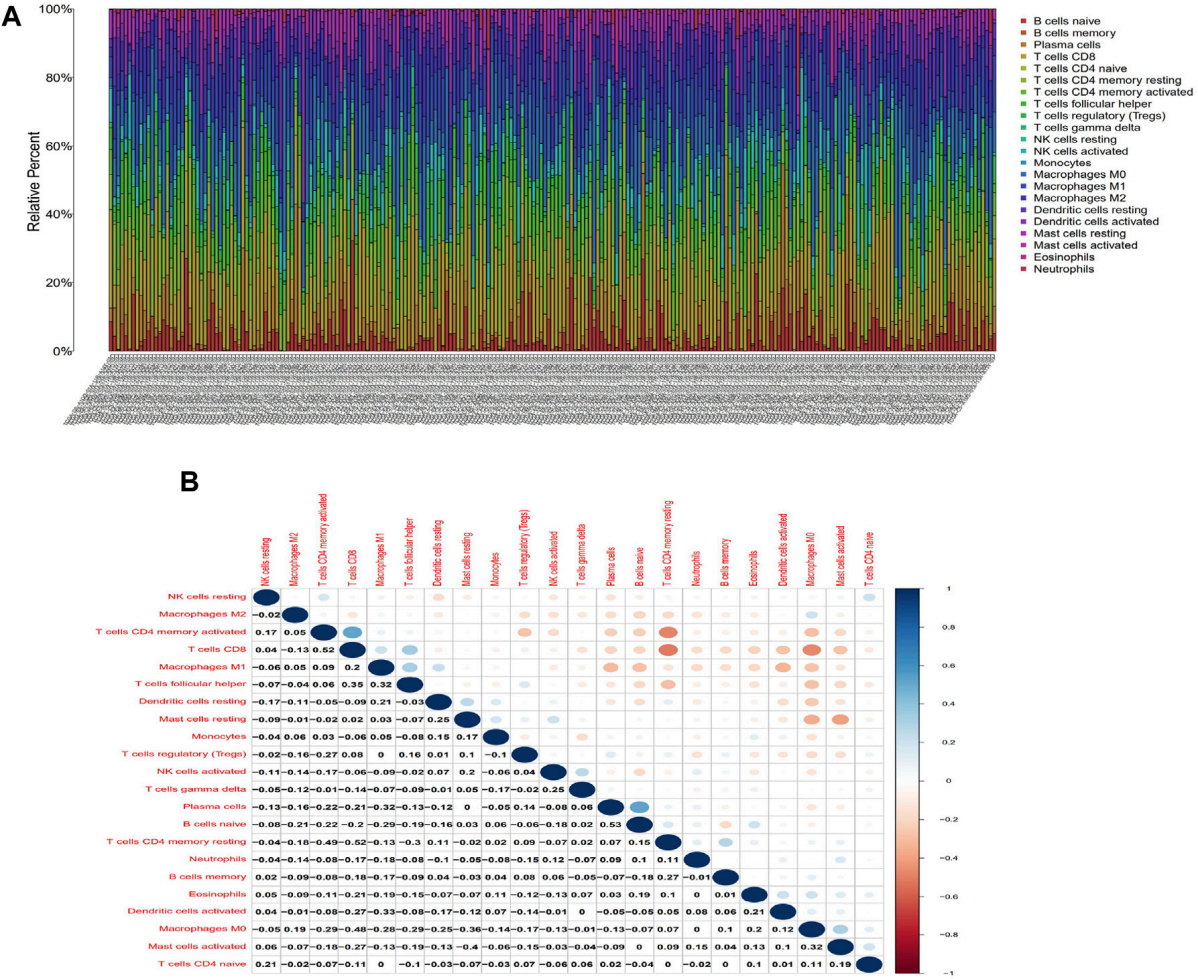
TIC profile in tumor samples and correlation analysis. **(A)**Barplot showing the proportion of 21 kinds of TICs in CESC with radiotherapy samples. Column names of plot were sample ID. **(B)**Heatmap showing the correlation between 21 kinds of TICs and numeric in each tiny box indicating the *p*-value of correlation between two kinds of cells. The shade of each tiny color box represented corresponding correlation value between two cells, and Pearson coefficient was used for significance test.

**FIGURE 8:**
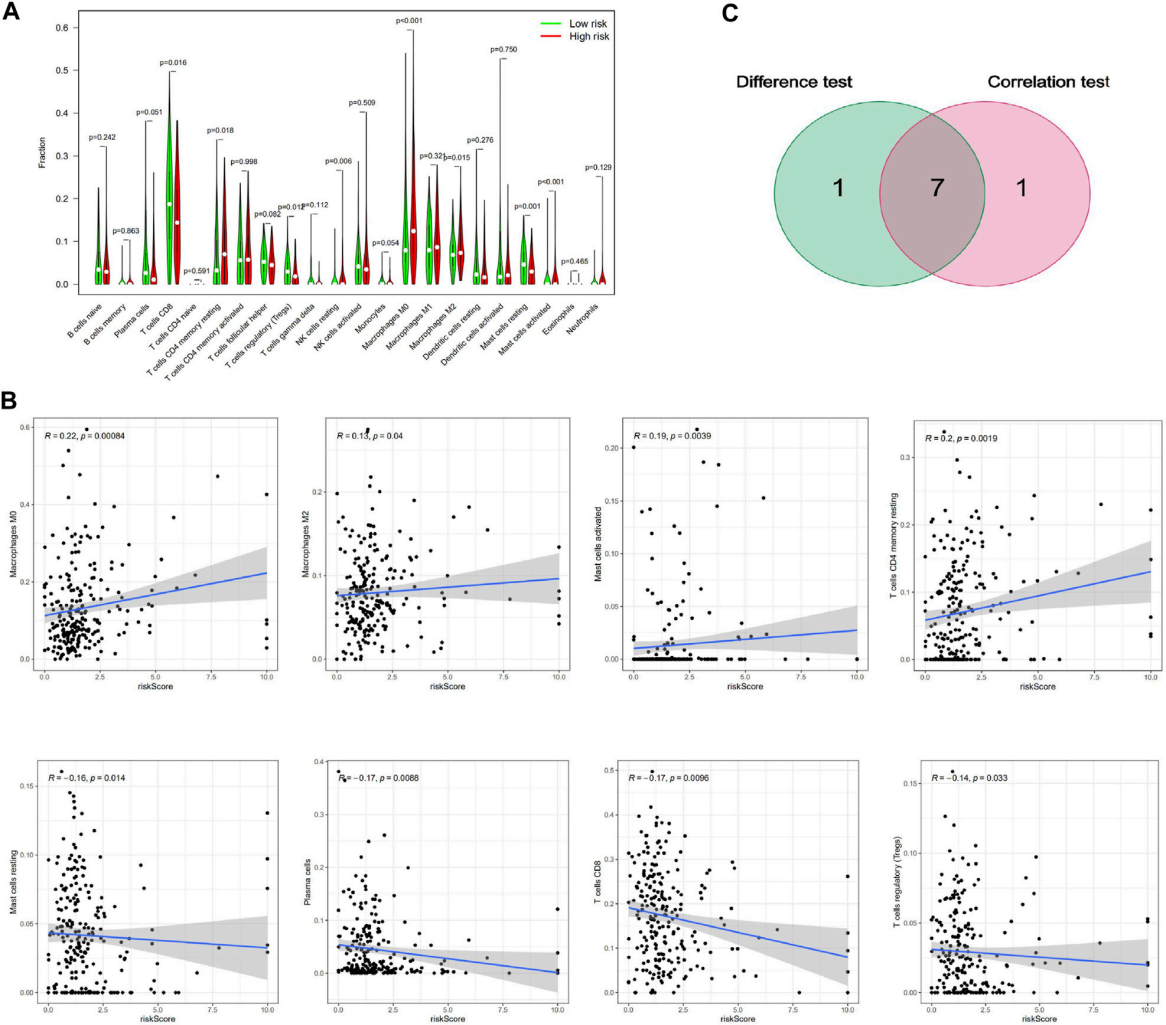
Correlation of TICs proportion with risk score system. **(A)**Violin plot showed the ratio differentiation of 21 kinds of immune cells between CESC with radiotherapy samples with low or high risk relative to the median of risk score level, and Wilcoxon rank sum was used for the significance test. **(B)**Scatter plot showed the correlation of 8 kinds of TICs proportion with the risk score system (*p* < 0.05). The blue line in each plot was fitted linear model indicating the proportion tropism of the immune cell along with risk score system, and Pearson coefficient was used for the correlation test. **(C)** Venn plot displayed seven kinds of TICs correlated with risk score system codetermined by difference and correlation tests displayed in violin and scatter plots, respectively.

To further verify the relationship between the risk score and the TME, we applied the CIBERSORT algorithm to determine the proportions of 22 kinds of immune cells in CESC samples with radiotherapy (Figures 7A,B). The components of immune cells in the cervical cancer with radiotherapy were explored that eight kinds of TICs (*p* < 0.05) (Figure 8A). The components of immune cells were showed that eight kinds of TICs were related to the risk score (*p* < 0.05) ((Figure 8B). Furthermore, a total of 7 kinds of immune cells were common to both the cervical cancer and risk-scoring system, as shown in the Venn diagram (Figure 8C). The impact of the risk score was further supported by these results regarding TME immune activity.

## 4 Discussion

Immune system dysfunction is an important mechanism by which cancer cells evade immune surveillance. Therefore, “immunonormalization” has become the basic guiding principle of immunotherapy (Sanmamed and Chen, 2018). Immunotherapy involves changes in genes, metabolism, inflammation, and tumor microenvironment, and the mechanisms are complex. Moreover, considering that atypical and even cancerous cells often coexist in the microenvironment with dense infiltration of inflammatory cells (Hanahan and Weinberg, 2011), it is challenging to study the changes in tumor genomics and the tissue microenvironment. In our study, we used DEGs to construct a risk scoring system that included 14 key genes to assess immune cell infiltration in the radioresistant cervical cancer microenvironment and to assess possible clinical outcomes. As expected, the high-risk groups had poorer overall survival and tumors that tended to persist after radiotherapy, which may indicate a poor prognosis.

The 14 key genes were all related to breast cancer in some way, especially SPRY4, MIEN1, AGT, and WT1. SPRY4 encoding proteins are inhibitors of the receptor-mediated MAPK signaling pathway, which was indicated in our previous KEGG enrichment results. SPRY4-IT1 expression is significantly related to breast cancer resistance to cisplatin, and increased SPRY4-IT1 expression is significantly associated with the lymph node metastasis rate and recurrence; in addition, patients with high spRY4-IT1 expression have lower OS and DFS rates than those with low expression (Zheng et al., 2020). Das Mrinal K also found in testicular germ cell tumors that SPRY4 expression (including mRNA, protein and SPRY4 - IT1) is significantly higher than that in normal testicular tissue and that small interfering RNA (siRNA)-mediated knockout of SPRY4 and SPRY4 - IT1 can obviously reduce the cell growth, migration, and invasion and significantly reduce the level of Akt phosphorylation; these results indicate that SPRY4 and SPRY4-IT1 may act as oncogenes through activation of the PI3K/Akt pathway, the same pathway that was enriched in the KEGG analysis (Das et al., 2018). MIEN1 is a primary regulator of tumor cell migration and invasion that is widely expressed as a membrane-fixed protein in various cancers (Kushwaha et al., 2019). Zhao et al. (Zhao et al., 2017)examined MIEN1 expression in 40 breast cancer and normal breast tissue samples using immunohistochemical staining, and the detection rate was 67.5% in breast cancer, which was significantly higher than that in normal breast tissue (0%, *p* < 0.05). The expression of MIEN1 was related to age, differentiation degree and lymph node metastasis. Kaplan-Meier survival analysis showed that the overall survival rate in patients with positive expression of MIEN1 protein was lower than that of those with negative expression of MIEN1 protein; in addition, overexpression of MIEN1 may contribute to the migration and invasion of breast cancer. In our study, MIEN1 expression was negatively correlated with radioresistance, possibly because our research subjects had cervical cancer treated with radiotherapy, and the expression of MIEN1 may change after radiotherapy. Angiotensinogen (AGT) is a rate-limiting substrate of the renin-angiotensin system (RAS). Sun et al. (Sun et al., 2019) found that AGT expression was inhibited by high glucose at both the transcriptional and translational levels, and high AGT remarkably suppressed proliferation, inhibited viability, and suppressed the migration/invasion of breast cancer cells, which was consistent with the results of our study. The WT1 gene encodes a type of zinc finger structural transcription factor and plays an important role in normal cell metabolism. The WT1 gene is specifically expressed in many solid and hematological tumor cells, and whether WT1 is a tumor suppressor or a proto-oncogene has always been controversial. Initial studies have shown that most specimens of human renal carcinoma have mutations in the WT1 gene, which are closely related to the occurrence, development and prognosis of leukemia (Shen et al., 2011). Therefore, the WT1 gene is considered a tumor suppressor gene. However, in breast cancer (Artibani et al., 2017), the WT1 gene is highly and specifically expressed, indicating that the known association between WT1 expression in breast cancer and poor prognosis is potentially due to poor chemotherapy response, so the WT1 gene can also be considered a proto-oncogene, which is similar to the results of our study. Yun et al. (Han et al., 2020)also showed that WT1 was significantly upregulated in human ovarian cancer tissues and was closely related to ovarian cancer type, differentiation degree and the International Federation of Gynecology and Obstetrics (FIGO) stage. WT1 may be a novel therapeutic target for improving the prognosis of ovarian cancer.

Similar to our research, Choi et al. (Choi et al., 2020) built a group of five DEG markers, namely, BCL2, HER2, CD133, CAIX and ERCC1, which can be used to predict the response to chemotherapy and prognosis of cervical cancer. The genes of their study are different to ours, and the reason may be that we studied immune-related genes. In contrast to previous studies, we first established and demonstrated a new prognostic system of immune-related genes that are differentially expressed in cervical cancer treated with radiotherapy.

The dynamic interaction of tumor cells and the microenvironment plays an important role in the proliferation, development and migration of tumors. Radiotherapy not only induces the death of immunogenic cancer cells but also acts as a risk factor to distribute immune signals that combat tumors, and ionizing radiation can trigger a tumor immune rejection mechanism (Formenti and Demaria, 2013). The main limitation of radiotherapy in activating antitumor immunity is the reduction in effector T cells (CD8^+^) (Park et al., 2015). Our study shows that CD8^+^ T cells are negatively related to the risk score, indicating that the number of CD8^+^ T cells decreases and the resistance to radiotherapy increases in patients with high-risk scores, which is the same result as the above research. M2 macrophages, myeloid-derived suppressor cells (MDSCs), immature dendritic cells, *etc.*, are abundant in the tumor microenvironment, which prevents the activation of T cells (Parker et al., 2015). Our study also showed that M2 macrophage infiltration is positively correlated with the risk score, which is associated with the radioresistance of the tumor and, in turn, the prognosis of the patient and the related biological functions; these results are consistent with those of the above research. Most studies have shown that the number and percentage of regulatory T cells (Tregs) with immunosuppressive effects after radiotherapy are higher than before (Persa et al., 2015), but some studies have shown that the inhibitory function of Tregs may be partially damaged by radiotherapy (Billiard et al., 2011). Our study showed a negative correlation between regulatory T cells and high-risk scoring systems, which is not consistent with most studies but can be similar to some studies (Billiard et al., 2011). However, the immune mechanisms involved need to be further studied. For the first time, our study showed that, after antigen exposure, the vast majority of the effector CD4^+^ T cells undergo apoptosis, part of the process which transforms them into memory T cells; this process is regulated by the antigen specificity of surviving cells and their transformation into a resting state (not activated) (Gasper et al., 2014). Further, our study shows that a high level of CD4^+^ memory T cell inactivation is positively correlated with a high-risk score, which is associated with radioresistance and a poor prognosis in patients; these results are consistent with the biological function of immune cells and the results of the above study.

Mast cells are innate immune cells that play a central control role in natural immunity, and as sentinel cells and effectors recruit cells, they regulate the host’s response to cancer and can be used to predict the prognosis of patients (Komi and Redegeld, 2020). Activated mast cells can produce and release angiogenic and inflammatory factors, exosomes, proteases, cytokines and chemokines. The correlation of mast cell infiltration with tumor prognosis varies among different tumors (Mao et al., 2018). Our study first indicated a positive correlation between activated mast cells and a high-risk score, which suggests that mast cell infiltration can be used to indirectly indicate prognosis and radioresistance. The possible mechanism is that activated mast cells secrete a series of factors through the interaction of immune cells and tumor cells to promote the formation of an immunosuppressive environment, which increases the activity of tumor cells and leads to a poor prognosis. In previous studies, mast cells were analyzed as a whole, but this study divided them into activated and unactivated cells, which may be useful in tumor prognosis study. In addition, we should expand the sample size to verify the results of the study and explore the concrete mechanism.

The importance of individual TICs in predicting the therapeutic effect has been the focus of research, and it is certain immune subgroups that play a major role rather than the overall scope of immune infiltration (Oliva et al., 2019). Classic immunohistochemical analysis is dependent on a marker to identify specific immune cell subgroups, unable to identify multiple subgroups, and is not effective for capturing certain cell phenotypes (such as activated lymphocytes and unactivated lymphocytes) (Chen et al., 2018). CIBERSORT uses a deconvolution algorithm and gene expression profiles to evaluate the relative percentage of different immune cell subgroups and overcomes the defects of the immunohistochemical methodology; in addition, it has been applied in colorectal cancer and kidney cancer (Xiong et al., 2018; Zhang et al., 2019). In this study, CIBERSORT was used to infer the percentages of the 22 types of immune cells in CESC with radiotherapy for the first time, and these values were used to comprehensively analyze the relationship between clinical features and the risk scoring system. However, our research also has certain limitations. First, the number of samples was small in our study, and larger cohorts and deeper sequencing methods are needed. Second, we focused on gene expression and ignored other events, such as mutation, methylation, and replication, which are also important factors in the progression of tumors and resistance to chemotherapy and radiotherapy. Finally, the molecular mechanism of the key gene, the relevant pathways and the role of immune cells in CESC with radioresistance are unknown and require further exploration. In summary, we built a novel risk scoring system with prognostic value that may predict the therapeutic effect of radiotherapy in CESC. Our findings suggest that the risk score is correlated with the tumor status after radiotherapy, and we suspect that high-risk patients may exhibit “immune escape” after radiotherapy. In addition, the key genes in our risk scoring system may provide new targets for immunotherapies for CESC that could be combined with radiotherapy and may provide new ideas to guide individualized treatment.

Our study has some limitations. First, the number of cases in the TCGA was low, especially radioresistance patients. Second, the reproducibility and function of the biomarkers and the related immune cell infiltration in CC should be further validated by prospective studies with larger sample sizes.

## 5 Conclusion

In conclusion, based on the TCGA database, we analyzed the immune characteristics of CESC treated with radiotherapy, 53 immune-related DEGs were identified. GO and KEGG analyses were applied to analyse the function and pathway of DEGs, and then 14 key immune-related genes established a novel risk scoring system that has predictive value. The high expression of WT1 and SPOUYT4 were associated with relapse, the high expression of AGT and MIEN1 were associated with nonrelapse. Analysis of the immune microenvironment indicated that M0 macrophages, M2 macrophages, activated mast cells and resting memory CD4^+^ T cells were positively correlated with the risk score. Therefore, our data might explore new biomarkers to predict sensitivity to radiotherapy in CC, and then for targeted therapy in CC with radiation.

## Data Availability

The original contributions presented in the study are included in the article/Supplementary Material, further inquiries can be directed to the corresponding authors.
