# Supplementary material for: A novel immune-related risk-scoring system associated with the prognosis and response of cervical cancer patients treated with radiation therapy
Source: Front Mol Biosci. 2023 Nov 10;10:1297774. doi: 10.3389/fmolb.2023.1297774 (PMC10667679; doi:10.3389/fmolb.2023.1297774)
Supplement: Supplementary file 2 [file DataSheet1.doc]

https://www.jianguoyun.com/p/DXpSt8oQ0dOWChi7zJ0FIAA
