# Supplementary material for: A novel immune-related risk-scoring system associated with the prognosis and response of cervical cancer patients treated with radiation therapy
Source: Front Mol Biosci. 2023 Nov 10;10:1297774. doi: 10.3389/fmolb.2023.1297774 (PMC10667679; doi:10.3389/fmolb.2023.1297774)
Supplement: Supplementary file 4 [file Table3.docx]

Table 3: Associations between expression of MIEN1 and clinicopathologic characteristics in cervical cancer

| Variable | Case | |  | Low MIEN1  expression | |  | High MIEN1  expression | |  | P value^a^ |
| --- | --- | --- | --- | --- | --- | --- | --- | --- | --- | --- |
|  | N | (%) |  | N | (%) |  | N | (%) |  |  |
| Age(years)^b^ |  |  |  |  |  |  |  |  |  |  |
| ≤59 | 41 | 59.42 |  | 14 | 56.00 |  | 27 | 61.36 |  | 0.663 |
| >59 | 28 | 40.58 |  | 11 | 44.00 |  | 17 | 38.64 |  |  |
| FIGO stage |  |  |  |  |  |  |  |  |  |  |
| I-II | 24 | 34.78 |  | 9 | 36.00 |  | 15 | 34.09 |  | 0.873 |
| III-IV | 45 | 65.22 |  | 16 | 64.00 |  | 29 | 65.91 |  |  |
| Histologic subtype |  |  |  |  |  |  |  |  |  |  |
| Squamous | 64 | 92.75 |  | 23 | 92.00 |  | 41 | 93.18 |  | 0.856 |
| Adenocarcinoma | 5 | 7.25 |  | 2 | 8.00 |  | 3 | 6.82 |  |  |
| Tumor grade |  |  |  |  |  |  |  |  |  |  |
| G1/G2 | 35 | 50.72 |  | 15 | 60.00 |  | 20 | 45.45 |  | 0.245 |
| G3 | 34 | 49.28 |  | 10 | 40.00 |  | 24 | 54.55 |  |  |
| Recurrence |  |  |  |  |  |  |  |  |  |  |
| No | 39 | 56.52 |  | 10 | 40.00 |  | 29 | 65.91 |  | 0.037^*^ |
| Yes | 30 | 43.48 |  | 15 | 60.00 |  | 15 | 34.09 |  |  |

Abbreviations: N = number; FIGO= Federation International of Gynecology and Obstetrics

^a^ Chi-square test, *p < 0.05.

^b^ Range 27-87 years, median 59 years
