# Supplementary material for: A novel immune-related risk-scoring system associated with the prognosis and response of cervical cancer patients treated with radiation therapy
Source: Front Mol Biosci. 2023 Nov 10;10:1297774. doi: 10.3389/fmolb.2023.1297774 (PMC10667679; doi:10.3389/fmolb.2023.1297774)
Supplement: Supplementary file 5 [file DataSheet2.docx]

**Supplementary Methods**

**RNA extraction and quantitative real-time PCR (qRT-PCR)**

Total RNA was extracted from frozen CC tissue utilizing Tirzol (TaKaRa, Japan) and then the RNA was then transcribed to cDNA using 5 X PrimeScript RT Master Mix (Takara, Japan). Real-time PCR was performed using TB Green^®^ Premix Ex Taq™ II (Tli RNaseH Plus) (Takara, Japan) and analyzed on a CFX connectTM (Bio-Rad, USA). The results were normalized to the expression of glyceraldehyde-3-phosphate dehydrogenase (GAPDH). The qRT-PCR data were analyzed and expressed relative to CT (cycle threshold) values. The qRT-PCR results were analyzed and showed as the fold change (2^-∆∆CT^). For the analysis of the expression, the levels were compared with the controls and converted to the fold change (2^-∆∆CT^). The quantitative PCR reaction for each sample was repeated in triplicate.

**Data collection**

These data were derived from the following resources available in the public domain: The CancerGenome Atlas (TCGA)- cervical squamous cell carcinoma and endocervical adenocarcinoma (CESC). https://portal.gdc.cancer.gov/ .

**R packages and and the corresponding results.**

|  | Molecular techniques | result |
| --- | --- | --- |
| 1. TCGA data merge and ID trans | Perl soft |  |
| 1. Analysis of variances and heatmap | “pheatamp” and “limma” package of R | 53 DEGs, Figure 2A/2C |
| 1. Volanco map | “dplyr”, “ggplot2”, “ggrepel” | Figure 2B/2D |
| 1. Venn | "venn" | Figure 2E |
| 1. GO | "clusterProfiler", "org.Hs.eg.db",  "enrichplot", "ggplot2" | Figure 3A |
| 1. KEGG | "clusterProfiler", "org.Hs.eg.db",  "enrichplot", "ggplot2" | Figure 3B |
| 1. riskplot | "pheatmap" | Figure 4B,4C |
| 1. ROC | "timeROC" | Figure 4D |
| 1. surivival | "survival","survminer" | Figure 4F |
| 1. Clinical correlation | "limma", "ggpubr" | Figure 4E |
| 1. riskplot | “limma”, “ggpubr” | Figure 5 |
| 1. CIBERSORT | ”e1071”, "BiocManager", "preprocessCore" |  |
| 1. immuneplot | "corrplot" | Figure7A,7B |
| 1. Differential analysis of immune cells | "limma","vioplot" | Figure 8A |
| 1. Correlation analysis between risk model and immune cells | “limma”, “ggplot2”, “ggpubr”, “ggExtra” | Figure8C |
|  | R packages | result |
| 1. TCGA data merge and ID trans | Perl soft |  |
| 1. Analysis of variances and heatmap | “pheatamp” and “limma” package of R | 53 DEGs, Figure 2A/2C |
| 1. Volanco map | “dplyr”, “ggplot2”, “ggrepel” | Figure 2B/2D |
| 1. Venn | "venn" | Figure 2E |
| 1. GO | "clusterProfiler", "org.Hs.eg.db",  "enrichplot", "ggplot2" | Figure 3A |
| 1. KEGG | "clusterProfiler", "org.Hs.eg.db",  "enrichplot", "ggplot2" | Figure 3B |
| 1. riskplot | "pheatmap" | Figure 4B,4C |
| 1. ROC | "timeROC" | Figure 4D |
| 1. surivival | "survival","survminer" | Figure 4F |
| 1. Clinical correlation | "limma", "ggpubr" | Figure 4E |
| 1. CIBERSORT | ”e1071”, "BiocManager", "preprocessCore" |  |
| 1. immuneplot | "corrplot" | Figure7A,7B |
| 1. Differential analysis of immune cells | "limma","vioplot" | Figure 8A |
| 1. Correlation analysis between risk model and immune cells | “limma”, “ggplot2”, “ggpubr”, “ggExtra” | Figure8C |
